# BusyBee Web: metagenomic data analysis by bootstrapped supervised binning and annotation

**DOI:** 10.1093/nar/gkx348

**Published:** 2017-05-02

**Authors:** Cedric C. Laczny, Christina Kiefer, Valentina Galata, Tobias Fehlmann, Christina Backes, Andreas Keller

**Affiliations:** Chair for Clinical Bioinformatics, Saarland University, Campus Building E2.1, 66123 Saarbrücken, Germany

## Abstract

Metagenomics-based studies of mixed microbial communities are impacting biotechnology, life sciences and medicine. Computational binning of metagenomic data is a powerful approach for the culture-independent recovery of population-resolved genomic sequences, i.e. from individual or closely related, constituent microorganisms. Existing binning solutions often require *a priori* characterized reference genomes and/or dedicated compute resources. Extending currently available reference-independent binning tools, we developed the BusyBee Web server for the automated deconvolution of metagenomic data into population-level genomic bins using assembled contigs (Illumina) or long reads (Pacific Biosciences, Oxford Nanopore Technologies). A reversible compression step as well as bootstrapped supervised binning enable quick turnaround times. The binning results are represented in interactive 2D scatterplots. Moreover, bin quality estimates, taxonomic annotations and annotations of antibiotic resistance genes are computed and visualized. Ground truth-based benchmarks of BusyBee Web demonstrate comparably high performance to state-of-the-art binning solutions for assembled contigs and markedly improved performance for long reads (median F1 scores: 70.02–95.21%). Furthermore, the applicability to real-world metagenomic datasets is shown. In conclusion, our reference-independent approach automatically bins assembled contigs or long reads, exhibits high sensitivity and precision, enables intuitive inspection of the results, and only requires FASTA-formatted input. The web-based application is freely accessible at: https://ccb-microbe.cs.uni-saarland.de/busybee.

## INTRODUCTION

Metagenomic sequencing, i.e. whole genome sequencing of DNA indiscriminately extracted from mixed microbial communities, was successfully used to study the taxonomic composition as well as the functional potential of environmental microbiomes (1–4). The independence of prior isolate culturing steps is often considered an advantage as this independence allows reduction in costs and time, as well as the potential to characterize microorganisms that, thus far, have resisted culturing attempts under artificial laboratory conditions (5,6). While metagenomic sequencing has been mostly used for basic research, its potential in clinical settings has been demonstrated recently (7,8). Moreover, third generation-sequencing technologies, e.g. from Pacific Biosciences (PacBio) or Oxford Nanopore Technologies (ONT), are emerging and enable the long read-based study of mixed microbial communities (9–11).

The recovery of genomic sequences resolved at the level of individual organisms (or populations of closely related organisms) from metagenomic sequencing data using computational solutions is termed ‘binning’. The current body of binning approaches can be roughly subdivided into (i) reference-dependent approaches and (ii) reference-independent approaches. Reference-dependent binning approaches are typically characterized by very low runtimes as well as high degrees of sensitivity and precision (12–16). However, these approaches, by design, perform best for sequences derived from organisms that are part of or are closely related to the references present in a database, and are challenged by genomic sequences derived from hitherto uncharacterized microorganisms. In contrast, reference-independent binning approaches do not rely on prior knowledge as they infer sequence cluster structures from the input data only (17–20) and are mostly based on sequence composition, with approaches relying on abundance co-variation across multiple samples emerging recently (21–24). Due to their reference independence, these approaches are of particular use for the analysis of environments with limited representations in the current reference genome databases, frequently allowing resolution of ‘unclassified’ sequences. However, reference-independent binning often requires substantial amounts of CPU hours, sequence lengths above a certain threshold, e.g. 1000 bp, and/or multiple, ideally independent, samples. While various binning web servers exist, these are mostly based on reference-dependent approaches (15,25–27), or require upfront computations which results in the need for dedicated computing resources and/or user training (28,29).

Here, we extend the currently available reference-independent binning tools by presenting the BusyBee Web server, a web application implementing bootstrapped supervised binning (BSB) of metagenomic sequencing datasets. Our binning approach combines unsupervised and supervised machine learning approaches by ‘bootstrapping’ the training data from the input rather than relying on reference databases. BusyBee Web only requires a single FASTA-formatted file as input and performs automated deconvolution of the sequences into population-resolved bins. During BSB, clusters are defined *de novo* on a subset of the sequences using an unsupervised approach (30–32). This step is followed by the training of a random forest-based classifier using the cluster labels as the response/dependent variables (supervised part). To further accelerate the binning, an optional ‘compression’ step is implemented in which data points are randomly sampled serving as representatives for their nearest neighbors (associates) during the unsupervised part (compression). The representatives as well as their associates are subsequently used during the supervised part in combination with the respective representatives’ *de novo* cluster labels (decompression). Thus, the training set size is increased compared to only using the randomly sampled, representative data points. Ultimately, every sequence (≥500 bp, by default) is assigned a label using the bootstrap-trained classifier, thereby defining the final set of bins. For inspection of the clustering/binning results, a 2D scatterplot of the data-inherent as well as the inferred structures is presented to the user. To complement this, estimates of bin quality, i.e. degrees of completeness, contamination and strain heterogeneity, are computed and visualized. Moreover, sequences are taxonomically annotated using Kraken and functional annotation of antibiotic resistance genes is performed. Because all of the binning and annotation steps are automatically executed by the web server transparently to the user, no dedicated computing resources or special user training is required. Furthermore, custom per-sequence annotations can be uploaded by the user, e.g. to highlight specific sequences of interest, and BusyBee Web offers the option to download the generated results should specialized downstream analyses be required, e.g. population-resolved annotation of KEGG pathways. Ground truth-based benchmarks comparing our BSB approach to state-of-the-art binning approaches are provided for assembled contigs (Illumina) and long reads (ONT). Moreover, the applicability of our web server for the analysis of real-world metagenomic datasets (Illumina or PacBio) is demonstrated. The BusyBee Web server is available free-to-use at https://ccb-microbe.cs.uni-saarland.de/busybee.

## IMPLEMENTATION

### Workflow

**Figure 1.**
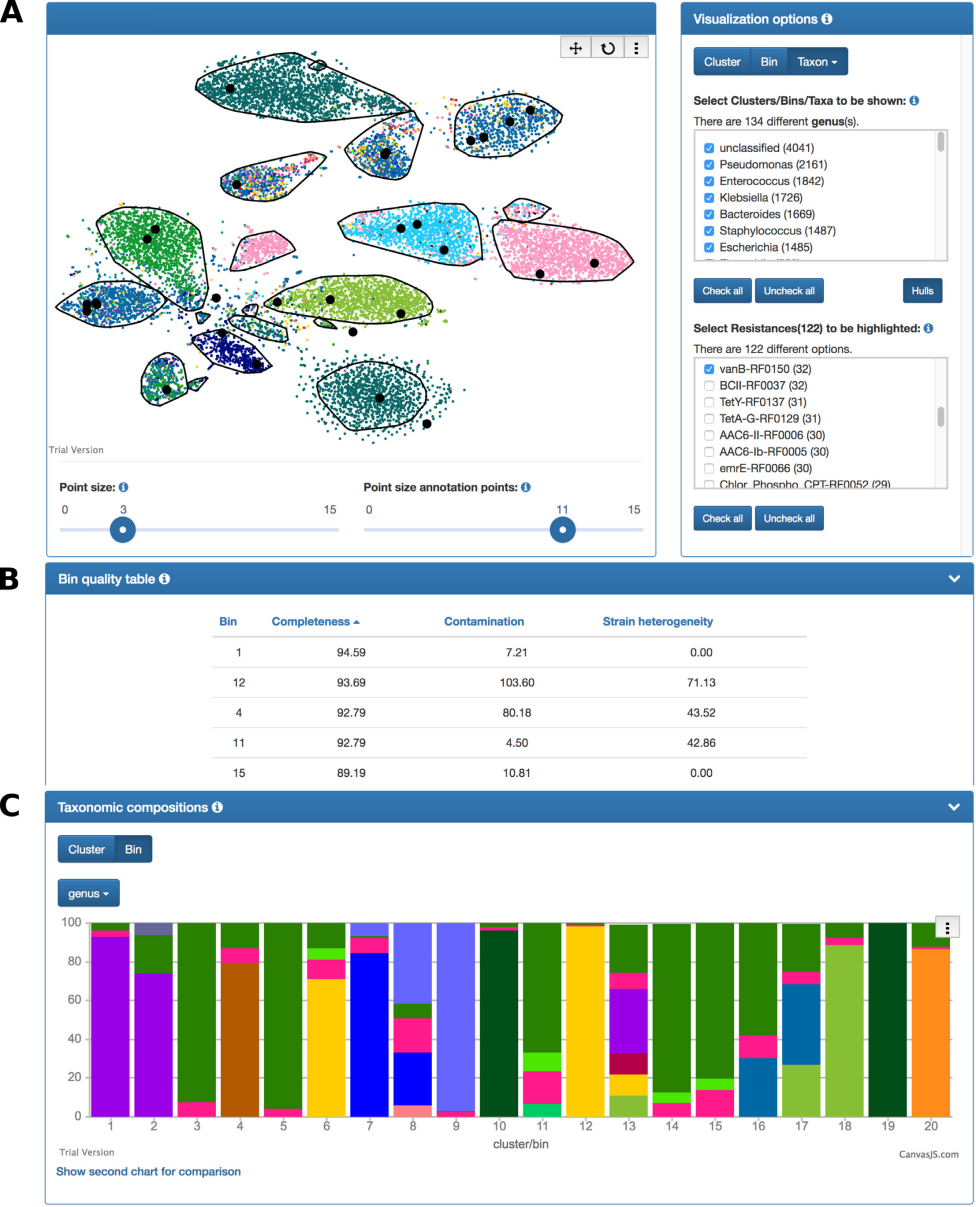
Overview of individual components of the BusyBee Web results page. (**A**) Input sequences are represented as individual points (according to the thresholds t_b_ and t_c_) in the 2D scatterplot. Convex hulls (black polygons) delineate the predicted clusters. If the optional taxonomic and functional annotations were enabled, taxon and antibiotic resistance-related information is shown to the right of the scatterplot. Individual clusters, bins or taxa can be shown or hidden and sequences encoding for specific antibiotic resistance genes can be highlighted using points of larger size and dark color, here, for the *vanB* gene. A left-click on a point reveals detailed information about the respective sequence, e.g. the taxonomic lineage or encoded antibiotic resistance genes. The user can pan and zoom the plot using the mouse, e.g. to focus on a region of interest, and point sizes are easily adjusted using sliders below the 2D scatterplot. (**B**) Bin quality estimates (completeness, contamination, strain heterogeneity) are provided as a sortable table, here, sorted by decreasing completeness. An excerpt representing the five most complete bins is shown. (**C**) The optional taxonomic compositions of the clusters/bins are shown as stacked bar charts. The taxonomic rank, e.g. genus, can be selected and a second chart can be shown to compare the compositions of the individual clusters/bins at different ranks, e.g. genus versus family.

When a new job is initiated, the user has to provide a FASTA-formatted file of nucleotide sequences, e.g. assembled contigs or long sequencing reads, as the only mandatory input. By default, population-level genomic bins are automatically defined by BSB of the input sequences followed by bin quality assessment. Moreover, BusyBee Web can optionally compute taxonomic annotations and annotations of antibiotic resistance genes. Custom, per-sequence annotations can also be provided by the user, e.g. to highlight specific sequences of interest. Importantly, as BSB is a reference-independent approach, population-level resolution is achieved even in the absence of taxonomically annotated reference genomes, e.g. for environments with limited representations in current reference databases. Robust default values for all BSB parameters are pre-set but can be adjusted by the user. Upon completion of all computational steps, the user can explore the results through interactive visualizations directly in the browser (HTML, JavaScript), e.g. to identify bins that are enriched for specific antibiotic resistance genes or bins that represent candidate hitherto uncharacterized microorganisms (Figure 1). Individual results can be shared using the unique job ID or the URL of the results page. Moreover, a zipped archive of the results can be downloaded for downstream processing. This archive includes the binning results (in particular, per-bin FASTA files), results from the bin quality assessment, as well as results from the optional taxonomic and functional annotation steps.

### Bootstrapped supervised binning

BSB is reference-independent and combines unsupervised as well as supervised machine learning approaches using genomic signatures in the form of oligonucleotide frequencies as the feature set (Supplementary Materials and Supplementary Figure S1). Supervised binning approaches are often equated with reference-dependent approaches, in particular, using reference genomes derived from microbial isolates for a priori training. However, more generally, a supervised machine learning approach uses training data to generate a model which is subsequently used for the classification of test data. The training data can be inferred from the input data as it is effectively done in our approach by first using an unsupervised machine learning approach (Supplementary Figure S1A). Accordingly, BSB can be seen as an extension of a classifier trained on a specific set of references, albeit bootstrapping the training data from the input data (19), rather than relying on previously characterized reference sequences (13,33). In brief, sequences are size-selected and separated into border points, cluster points and remaining points according to the user-specified parameters (border points sequence length threshold, t_b_ and the cluster points sequence length threshold, t_c_). Each sequence is then represented by its genomic signature, using pentanucleotide frequencies (default). Optionally, the border points and the cluster points are ‘compressed’ which will reduce the runtimes of both, the 2D embedding and the automated clustering. Following this, the 2D embedding is computed (20,32). Subsequently, automated clustering (30,31) is performed on the cluster points only, while the border points are supposed to help push individual clusters further apart and, thus, improve the automated segregation into distinct sequence clusters (Supplementary Figure S1B). The clustering information is used to train a random forest-based classifier, which predicts cluster assignments for the input sequences (≥500 bp; default), thereby defining the final set of bins (Supplementary Figure S1). In this context, it is important to highlight the difference between a ‘cluster’ and a ‘bin’. While both represent sequence sets, a ‘cluster’ is an intermediate sequence set and a ‘bin’ is a final sequence set. Consequently, a cluster may represent only a limited fraction of a population-level genome, while a bin tries to maximize the recovery of genomic information derived from the respective population.

While generally robust default parameters are provided in BusyBee Web, the user might need to specify custom settings based on the characteristics of the input data. For example, given highly fragmented assemblies or datasets with narrow sequence length distributions, the border points sequence length threshold, t_b_ and the cluster points sequence length threshold, t_c_, may be set to equal values, e.g. decreasing t_c_ to the value of t_b_. The ‘minPts’ parameter value may be decreased to allow the identification of small-sized clusters. In this context, if the degree of compression is set too high and the ‘minPts’ parameter is not decreased accordingly, clusters might be missed. This typically becomes apparent as distinct groups of points in the 2D visualization lacking a convex hull, i.e. not delimited by a polygon. Moreover, increasing the minimum sequence length can avoid the annotation of short sequences and thus decrease the fraction of incomplete genes. Detailed parameter descriptions are provided in the Supplementary Materials and as online tooltips.

### Annotations

#### Taxonomic annotation

Kraken (v0.10.5-beta) in combination with the Minikraken database, i.e. a reduced-size database constructed from complete bacterial, archaeal and viral genomes in RefSeq as of 8 December 2014 (https://ccb.jhu.edu/software/kraken/dl/minikraken.tgz), is used to compute taxonomic annotations for the input sequences (14). The reduced-size database was chosen due to its low memory requirements. However, the integration of a larger database is possible in the future to increase the sensitivity of the taxonomic annotations.

#### Annotation of antibiotic resistance genes

Prokka (v1.11) with the ‘—fast’ option is used for gene (CDS) calling (34,35) on all input sequences. The translated CDS sequences are then searched against the ResFams collection of antibiotic resistance genes (36) using hmmsearch from HMMER (v3.1b2; http://hmmer.janelia.org/).

#### Custom annotations

Custom annotations can be uploaded to highlight individual sequences or sequence sets. The former can, for example, be used for sequences encoding genes with a particular function and the latter for sequences annotated with a custom reference genome database or characterized according to their genomic or transcriptomic fold-coverage, or ratio of both (high/medium/low) (37). To enable this option, a tab-separated text file containing the sequence ID in the first column and the respective annotation in the second column should be provided by the user.

#### Bin quality assessment

CheckM (v1.0.7) is used to evaluate the quality (degrees of completeness, contamination and strain heterogeneity) of the individual bins using a custom set of marker genes (‘essential genes’) (38–40). The default memory requirement of CheckM (≥16 GB RAM) is prohibitive for use in a web application serving multiple users concurrently. Hence, the use of a custom set of marker genes which reduces the memory requirements of CheckM considerably by bypassing the reference genome-tree placement. While the currently implemented custom set is bacteria-specific, extended sets can be integrated into BusyBee Web in the future to represent microorganisms from other domains, e.g. archaea.

### Representation of the results

**Figure 2.**
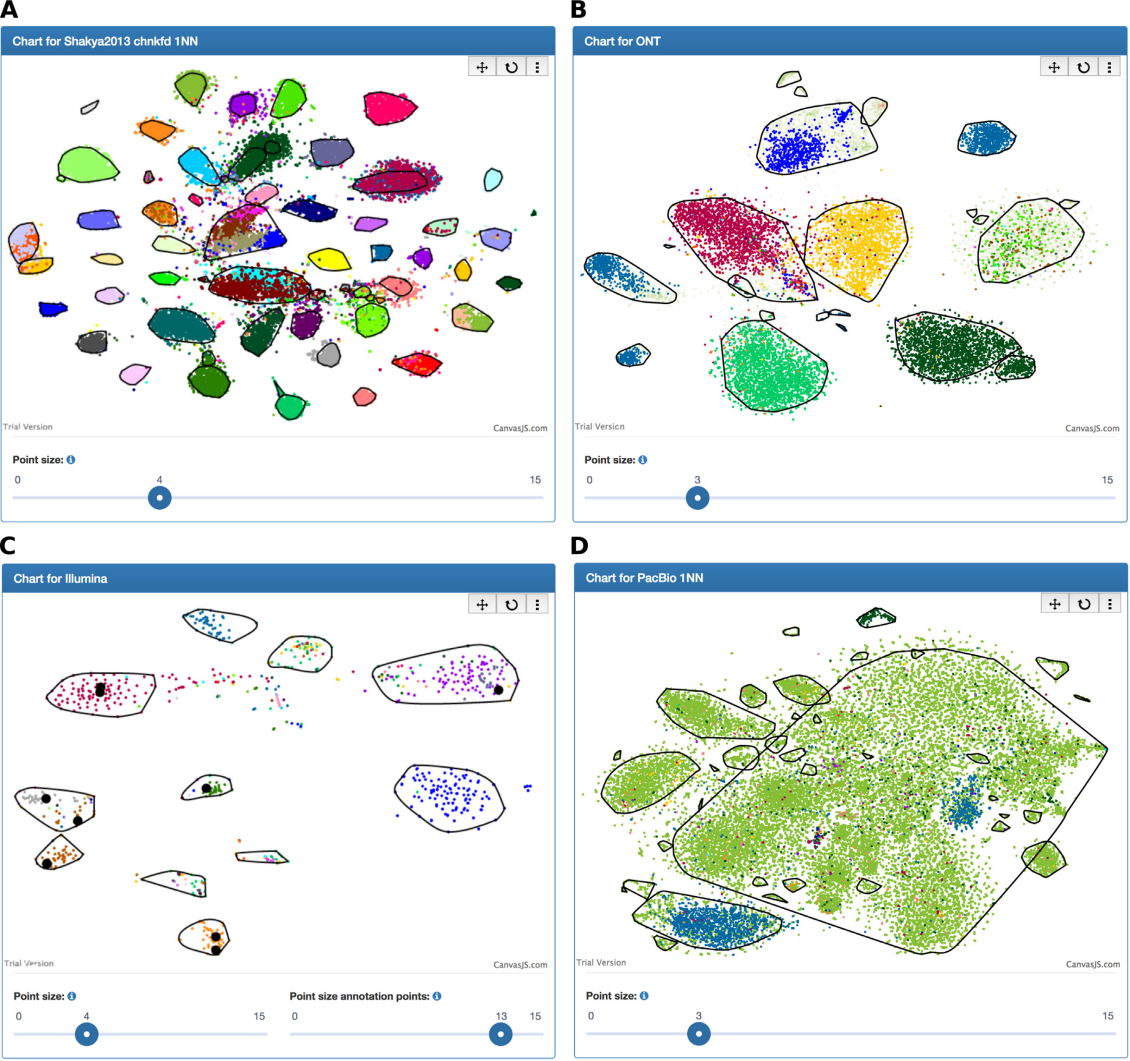
Screenshots of the interactive scatterplots for (**A**) ground truth-based Illumina (Shakya2013), (**B**) ground truth-based ONT, (**C**) small-scale Illumina and (**D**) PacBio metagenomic data. (A) A compression of 1 (‘1NN’) as well as sequence chunks (3 kbp chunk-length) derived from the full-length contigs were used. (B) Only sequences with species-level taxonomic assignments are shown. (C) Sequences encoding for class A CTX-M beta-lactamases (CTXM-RF0059) are highlighted. (D) A compression of 1 (‘1NN’) was used. The convex hulls (black polygons) delineate the individual sequence clusters. Descriptions at the top of each plot represent job names; if none is specified, a unique job ID is shown. Colors are based on species-level taxonomic assignments.

BusyBee Web provides interactive visualizations of the results (Figures 1 and 2). The automated clustering/binning results are represented as a 2D scatterplot, with individual points colored according to their assignment (cluster, bin or noise; Supplementary Figure S1) and each point representing an input sequence with length ≥t_b_. Convex hulls are additionally plotted to help in delineating the individual clusters. Suboptimal automatically defined clusters can thus be identified visually, e.g. distinct clusters which have been artificially joined. Clicking on individual points provides detailed information on the point's optional annotations, i.e. the predicted taxonomy and antibiotic resistance genes encoded by the respective sequence. Moreover, the user can change the size of the points as well as pan and zoom the plot. Individual clusters, bins or taxonomic groups (e.g. at the genus-level or at the species-level) can be selected. Unselected points are plotted with reduced opacity. Similarly, groups of sequences encoding specific antibiotic resistance genes or sharing individual, user-provided annotation can be shown or hidden. The number of contigs per cluster and per bin is shown as a bar chart. This allows the user to see how many sequences represented a cluster during the training phase and how many sequences were assigned to a bin by the trained classifier. Furthermore, bin quality estimates (completeness, contamination, strain heterogeneity) are displayed as a bar chart and as a sortable table. The taxonomic compositions per cluster and per bin are shown as stacked percent bar charts and a second chart of taxonomic compositions can be opened, thereby allowing the comparison of cluster/bin taxonomic compositions at different taxonomic ranks, e.g. at the family-level and the genus-level. A zipped archive of the results, including per-bin FASTA-formatted files and per-sequence taxonomic annotations among others, can be downloaded.

### Metagenomic datasets to evaluate BusyBee Web

Two metagenomic datasets of short read-assembled contigs (Shakya2013 (41), Gregor2016 (13); Illumina) as well as one raw, long read sequencing-based dataset (ONT) representing microbial communities of known composition (42–48) (Table 1, Supplementary Materials and Supplementary Table S1), i.e. representing ground truth data, were used to quantitatively assess the performance of our BSB approach and to compare it against two state-of-the-art binning approaches, MaxBin2 and MetaBAT (23,24).

**Table 1.** BusyBee Web runtimes reported in minutes for the herein studied ground truth and real-world datasets

		# sequences	Total length [bp]	Binning runtime [min]	Total runtime [min]
Ground truth	Shakya2013	24 974	179 063 212	8	30
	Gregor2016	14 393	142 556 476	6	23
	ONT	21 000	97 715 136	11	20
Real-world	Small-scale Illumina	859	50 964 782	1	6
	Large-scale Illumina^‡^	133 149	399 132 179	28	75
	PacBio^†^	71 029	93 937 106	18	27

Runtimes were determined manually based on the progress interface in the browser and were rounded to the next full minute. The minimum sequence length threshold was 1 kbp for the large-scale Illumina dataset and 500 bp for the other datasets.

^†^Compression of 1 was used.

^‡^Compression of 2 was used.

Three additional metagenomic datasets were used to demonstrate the versatility of the BusyBee Web server: two Illumina-based datasets (small-scale (49), large-scale (39)) and a PacBio-based dataset (10) were used as originally provided (Table 1). The PacBio dataset consisted of Circular Consensus Sequences (CCS) which provide increased sequence quality by repeatedly sequencing the same molecule (50), thereby correcting for sequence errors. However, no additional error correction nor assembly were performed on the CCS reads.

## RESULTS AND DISCUSSION

To cover the heterogeneity of currently available sequencing technologies, we applied BusyBee Web to Illumina-, PacBio- and ONT-based sequencing data. Moreover, we compared the binning performance of BusyBee Web against MaxBin2 and MetaBAT on three ground truth datasets.

### Ground truth-based evaluation of BSB

We used two Illumina-based (Shakya2013, Gregor2016) and one ONT-based metagenomic dataset of defined composition to evaluate our BSB approach (Table 1). The numbers of bins inferred by BSB were 45/38 (Shakya2013/Gregor2016), with 58/45 expected species (Supplementary Notes and Supplementary Tables S2–5). Normalization of the cluster density by using sequence chunks (3 kbp chunk-length) derived from the full-length contigs (49,51) resulted in 60/50 bins. Moreover, the sensitivity, precision and F1 values were substantially increased (Supplementary Tables S3 and 5). For example, the median precision value was almost 20% higher using sequence chunks (91.49%; Figure 2A) instead of the full-length contigs (71.99%; Supplementary Figure S2), and the median F1 score increased to 90.09 from 70.02% for the Shakya2013 dataset.

For the ONT-based ground truth data (t_b_ = t_c_ = 500 bp), our approach reported 23 bins, with the large bins representing the six constituent bacterial organisms (Figure 2B). The influenza A virus-derived sequences formed at least three major bins. The bin quality assessment yielded no representative results which may be due to the increased error-rate of the raw, nanopore sequencing-based reads (52–54) and the use of read subsamples for this dataset (Supplementary Table S6). About 31.91% (6701/21 000) of the sequences remained unclassified at the phylum-level, which is likely due to their increased error-rate. Nevertheless, BusyBee Web created representative bins for all the included isolates resulting in mean/median F1 scores of 89.00/92.66% (Supplementary Table S7). Processing only the influenza A virus-derived (subsampled) sequences revealed eight bins (Supplementary Figure S3). While an in-depth study of the individual bins was beyond the scope of the current work, this serves as an example of using BusyBee Web to inspect microbial isolate-derived genomic sequences or bins generated by a complementary binning tool for the presence of multiple sequence clusters, e.g. due to multiple chromosomes or possible contaminations.

### Benchmarking against existing binning tools using ground truth data

We compared the results of our BSB approach to two state-of-the-art approaches, MaxBin2 and MetaBAT. These tools were selected as they both support single sample-based binning. As described above, BSB identified 45/38 (Shakya2013/Gregor2016) bins for the Illumina-based ground truth data. In comparison, MetaBAT produced 63/41 bins and MaxBin2 produced 58 bins for the Shakya2013 data but was omitted for the Gregor2016 data due to missing coverage information. While MetaBAT typically had high precision values for both Illumina-based ground truth datasets, the sensitivity was often low (Supplementary Tables S3 and 5). MaxBin2 had higher mean/median sensitivity compared to MetaBAT on the Shakya2013 data, yet had low mean/median precision (59.76/57.09%). Using our BSB approach, the highest median F1 scores were reached with 90.09 and 95.21% for the Shakya2013 and Gregor2016 chunked datasets, respectively (Supplementary Notes).

For the ONT data, MetaBAT and MaxBin2 returned 18 and 2 bins, resulting in mean/median F1 scores of 58.35/56.92% and 40.26/34.56%, respectively (Supplementary Table S7). MaxBin2 and MetaBAT use an empirically determined probability distribution for the tetranucleotide frequency distances (23,24). This distribution is learned *a priori* on high quality reference genomes. The increased sequence error rate of third generation-sequencing data is likely to negatively impact the distance calculations, i.e. two sequences might have larger tetranucleotide frequency distances despite being derived from the same genome. Consequently, this is likely to have negatively affected the binning performance in MaxBin2 and MetaBAT. Moreover, coverage values are a mandatory input to MaxBin2, yet were unavailable for the unassembled, long read ONT data. Hence, surrogate, unit coverage values were used for MaxBin2 while MetaBAT defaulted to coverage-free binning using tetranucleotide frequencies. While coverage information provides important information for binning and bin refinement (1,21,23,24,51), an initial assembly is required onto which reads can be mapped to compute the fold-coverage of the assembled contigs. However, if the reads are sufficiently long, e.g. >1000 bp, the binning can be performed prior to the assembly, thereby facilitating population-level assemblies. Accordingly, our BSB approach can be used to pre-partition raw, metagenomic, long reads, thus enabling a ‘divide and conquer’ approach.

### Real-world metagenomes

For the small-scale Illumina-based dataset (Figure 2C and Table 1), a total of 11 bins was identified with 7 near-complete bins (≥90% complete) and 4 partially complete bins (≥50%). A total of 5 of the 11 bins had contamination degrees ≥20% with 2 of the 5 bins showing high degrees of strain heterogeneity (≥80%). This indicates that sequences derived from closely related organisms were grouped together while sequences derived from more distantly related organisms were separated. Class A CTX-M β-lactamases were highlighted to demonstrate the antibiotic resistance gene annotation functionality (Figure 2C). A total of 6 of the 11 bins were found to contain sequences encoding for the respective genes. For the large-scale Illumina-based dataset (Table 1) a compression of 2 was used, resulting in 51 bins (Supplementary Figure S6) of which 11 were ≥90% complete and 33 were ≥50% complete. The average/median degrees of completeness, contamination and strain heterogeneity were found to be 62.36/74.77%, 85.96/11.71% and 22.75/9.81%, respectively.

For the analysis of the PacBio dataset (Table 1), a compression of 1 was used and the border points and cluster points thresholds were set to 500 bp due to the small average read length of 1319 bp. A large bin (bin number 1), including sub-structures that were not resolved by the automatic clustering step, dominated the results visualization (Figure 2D; center of the scatterplot). However, the interactive visualization in BusyBee Web enables the user to easily identify suspect bins, e.g. bins with suboptimal automated deconvolution. A detailed inspection and refinement (55–57) of the suspect bins can be subsequently performed using a user-driven binning approach, such as anvi'o or VizBin (29,32). Overall, the bins were less complete compared to the Illumina-based dataset (3 bins ≥50%). It should be noted that the Illumina-based data was derived from the sequencing of 11 samples (∼2.4 Gbp per sample) (49), while the PacBio-based data consisted of 94 Mbp of CCS reads derived from 8 flow cells (10). About 90.82% (32 255/35 515; after compression) of the sequences could not be classified at the phylum level using Kraken in combination with the Minikraken database. However, our BSB approach assigned 91.16% (64 753/71 029) of the total of sequences to the five largest bins.

The total runtimes for the herein studied datasets were between 6 and 75 min (Table 1) and the BSB step required <30 min for the largest dataset (133 149 sequences; 399 132 179 bp). While the taxonomic annotation step is fast (below 5 min for the large-scale Illumina dataset), a considerable and highly variable proportion of the runtime is spent by the bin quality control. The high variability might be explained by varying amounts of identified single copy marker genes.

## CONCLUSION

Metagenomic sequencing has become a widely used approach for the culture-independent study of mixed microbial communities and is often coupled with *in silico* deconvolution of metagenomic sequence fragments into population-resolved genomic bins (‘binning’) in order to study the constituent micro-organisms at an organismal level. While several binning approaches have been developed, they mostly require previously characterized references, substantial computing resources and/or prior user training. Here, we presented the BusyBee Web server for the automated, reference-independent binning and visualization of metagenomic data in the form of assembled contigs (Illumina) or long reads (PacBio, ONT). The web-based interactive representations, including a 2D embedding of genomic signatures, bin quality assessment using single copy marker genes and optional taxonomic assignments, allow for intuitive inspection of the results. This can help the user to build confidence in the individual bins while simultaneously facilitating the identification of sequence groups requiring special attention. In addition, automatically generated annotations of antibiotic resistance gene-encoding sequences or user-provided, per-sequence annotations are optionally overlaid on the 2D embeddings, e.g. with the former allowing to identify population-level genomes enriched for genes possibly conveying specific antibiotic resistances. The only mandatory input consists of a FASTA-formatted nucleotide sequence file and all computations are performed online and transparently to the user. Hence, no special user training, software installation or dedicated computing resources are required and individual results can easily be shared via the web. Moreover, BusyBee Web was evaluated on ground truth and real-world metagenomic data, with the ground truth-based benchmarks demonstrating comparable performance to state-of-the-art binning approaches for assembled contigs and markedly improved performance for long reads when using our approach. Overall, BusyBee Web facilitates population-level resolved analyses of metagenomic data, thereby being of service for the study of mixed microbial communities derived from various environments and sequencing technologies.

## Supplementary Material

Supplementary Data
