# Supplementary material for: BusyBee Web: metagenomic data analysis by bootstrapped supervised binning and annotation
Source: Nucleic Acids Res. 2017 May 2;45(Web Server issue):W171–9. doi: 10.1093/nar/gkx348 (PMC5570254; doi:10.1093/nar/gkx348)
Supplement: Supplementary Data [file gkx348_Supp.pdf]

BusyBee Web: metagenomic data analysis by bootstrapped supervised binning and annotation

Cedric C. Laczny[1,*] (cedric.laczny@ccb.uni-saarland.de), Christina Kiefer[1] (s9crkief@stud.uni-saarland.de), Valentina Galata[1] (valentina.galata@uni-saarland.de), Tobias Fehlmann[1] (tobias.fehlmann@ccb.uni-saarland.de), Christina Backes[1] (c.backes@mx.uni-saarland.de), Andreas Keller[1] (andreas.keller@ccb.uni-saarland.de)

[1] Chair for Clinical Bioinformatics, Saarland University, Campus Building E2.1, 66123 Saarbrücken, Germany

* To whom correspondence should be addressed. Tel: +49 681 302 68610 Email: cedric.laczny@ccb.uni-saarland.de

The authors wish it to be known that, in their opinion, the first two (2) authors should be regarded as joint First Authors

Supplementary Materials

Bootstrapped supervised binning

Genomic signatures in the form of oligonucleotide frequencies (default: k = 5) are computed for all sequences equal to or exceeding a minimum sequence length (default: 500 bp). The frequencies are standardized (centered and scaled to unit-variance; default). Alternatively, the frequencies may be centered log ratio-transformed. Following the transformation, the dimensionality is reduced using PCA via SVD from the original 512D (for k = 5, accounting for reverse complements and palindromes) to 50D (default) (1) using the “svds” function from the RSpectra package (2). This reduces the memory and runtime requirement considerably.

The transformed oligonucleotide frequencies are separated into three disjoint point subsets: 1) cluster points, 2) border points, and 3) remaining points, according to the cluster points sequence length threshold (t_c, default: >= 2,000 bp), border points sequence length threshold (t_b, default: >= 1,000 bp and < t_c), and remaining points sequence length threshold (t_r, default: >= 500 bp and < t_b), respectively (Supplementary Figure 1). Cluster points and border points are embedded into 2D using BH-SNE (1, 3, 4) implemented in the Rtsne package (5) (Supplementary Figure 1 A). It is possible to perform the clustering on all the points by setting all thresholds to be equal ($t_c = t_b = t_r$). However, this will negatively affect the runtime as many more points will be embedded and clustered.

Cluster points, as indicated by the name, are points that represent sequences which will be used during the automated clustering step using DB-SCAN (6). Due to their length (default > 2,000 bp) their genomic signatures are less variable than for shorter sequences, i.e., the variation in the high dimensional (signature) space as well as in the embedded (2D) space are reduced. Border points, in contrast, are only used during the embedding but not during the clustering (Supplementary Figure 1 B). The motivation here is that border points are supposed to help push individual clusters further apart, i.e., increase the separation of individual clusters, and, thus, improve the automated segregation into distinct sequence clusters. The “minPts” parameter value for DB-SCAN is set to 30 by default (dbscan package (7)). The “eps” parameter value is determined automatically using the

“changepoint” package and the “cpt.var()” function (8). DB-SCAN automatically determines the number of clusters (N) based on the “minPts” and the “eps” parameter.
After the clustering, a random forest model-based classifier is trained using the PCA-reduced oligonucleotide frequencies, i.e., in 50D (default), as the predictor variables and the cluster assignment as the response variable as input to the randomForest-function of the randomForest R-package (9) with a default number of 100 trees (ntree = 100). The trained model is subsequently used to predict bin assignments for all sequences greater than a minimal length threshold (default: 500 bp; Supplementary Figure 1). To differentiate between the cluster labels, which are obtained from the application of DB-SCAN, and the classification labels, which are predicted from the trained model, we refer to sequence groups in the final output of BusyBee as “bins”.
Optionally, the user may specify a probability threshold (default: 0). If the maximum predicted probability of a sequence belonging to a bin is below this threshold, the sequence will be reported as bin-noise, i.e., bin “0”. The size of this bin will depend upon the chosen probability threshold, with a higher threshold leading to a larger noise bin and a lower threshold leading to a smaller, potentially empty, noise bin.

Compression

While disabled by default, the bootstrapped supervised binning can be executed on a “compressed” set of points to further accelerate the computations. The compression is achieved by randomly selecting a subset of points from the cluster points and from the border points as representatives. For each of these representatives, the nearest-neighbor among the remaining points (associate point) is computed using the nn2() function from the RANN R-package (10). The representative points are then embedded and clustered as described above. Upon classifier-training, the representative points as well as their associate points are used, with the associate points being assigned the same cluster label as their respective representative point (decompression).
Similar to the uncompressed setting, once the classifier is trained, all points (sequences) are assigned to a bin. The larger the number, n, of nearest neighbors, the stronger the compression ratio and the sparser the set of representative points. As an example, for n = 1, about half of the cluster and border points is used as representatives, while for n = 2, about one third of the cluster and border points is used as representatives.

Ground truth data

Shakya2013. This dataset comprises 16 archaeal and 48 bacterial strains (11) and was downloaded from the NCBI’s SRA (SRR606249). The bbduk.sh script (BBtools-36.49) was used for quality filtering and trimming (“bbduk.sh in1=IN_1.fq.gz in2=IN _2.fq.gz overwrite=true out1=OUT_1.fq.gz out2=OUT_2.fq.gz ref=resources/adapters.fa ktrim=r k=23 mink=11 hdist=1 tpe tbo qtrim=rl ftm=5 trimq=20 minlen=100 maq=20”) and only the paired-end reads were kept. The trimmed paired-end reads were deduplicated using the FastUniq tool (12) using default options. The quality of the raw and the preprocessed datasets was evaluated using FastQC (http://www.bioinformatics.babraham.ac.uk/projects/fastqc/). The trimmed and deduplicated paired-

end reads were assembled using SPAdes-3.9.0 (“spades.py -1 IN_1.trim.uniq.fq.gz -2 IN_2.trim.uniq.fq.gz -o out_dir -t 30 -k 21,33,55,77 --careful”) (13). Contigs with a length < 500 bp were discarded. The originating organism for each contig was determined based on a Kraken search against the Minikraken database (https://ccb.jhu.edu/software/kraken/dl/minikraken.tgz) (14). The following species’ genomes were missing from the Minikraken database and were ignored: *Desulfovibrio piger*, *Sulfitobacter* sp. EE-36, *Sulfitobacter* sp. NAS-14.1, and *Sulfurihydrogenibium yellowstonense* strain SS-5. Strain-level taxa were collapsed to their respective species level, e.g., *Methanococcus maripaludis* C5 and *Methanococcus maripaludis* S2 were represented as *Methanococcus maripaludis.* Contigs with no assignment on the species level were ignored. A total of 58 expected species remained after removal and collapsing of taxa.

Gregor2016. This dataset comprises 47 strains from 45 different species (37 different genera) (15). In brief, metagenomic sequencing reads were created using a custom read simulator and assembled using Metassembler which was run with different k-mer sizes ranging between 19 and 75. The resulting contig sets were subsequently merged with Minimus2, contigs <= 1,000 bp were discarded, and the reference taxonomic identifiers for each remaining contig were recovered based on BLAST searches. The respective data was downloaded from https://github.com/algbioi/datasets/blob/master/simulated_dataset_uniform.7z.

Oxford Nanopore Technologies (ONT). This dataset was created by randomly subsampling the sequencing runs of a total of seven bacterial and viral isolates (16–22) (Supplementary Table 1) to 3,000 reads per isolate and pooling the subsampled reads. The data was used as-is and was not error-corrected or assembled.

Comparison to existing tools

MaxBin2-2.2.1 (23) and MetaBAT-0.32.4 (24) were used as state-of-the-art reference-free binning tools. MetaBAT was evaluated on all three ground truth datasets, i.e., on assembled contigs, as well as on unassembled, long reads. MaxBin2 was evaluated on the Shakya2016 and on the ONT data. All tools were run using a single thread. For MaxBin2, the intermediate results were preserved (“-preserve_intermediate”) and the minimum contig length was set to 500 (“-min_contig_length 500”). For MetaBAT, the minimal contig size was set to 1,500 bp (”-m 1500”) as per the tool’s suggested minimum. Unbinned sequences were preserved (“--unbinned”) and the “—verysensitive” option was used.

Shakya2013: The first pair of the quality-filtered and trimmed reads was used to compute the coverage in MaxBin2 as there was no option to specify paired-end sequencing data. The resulting (intermediate) SAM file was converted to a sorted BAM file using samtools-0.1.19 (25). This BAM file was used as input to the jgi_summarize_bam_contig_depths script from MetaBAT with the script’s output representing the abundance file (“-a”).

ONT: As the ONT data consisted of unassembled, long reads, no coverage information was computed. Because MaxBin2 requires an abundance file, a custom file with all coverages set to “1” was generated and used as input. For MetaBAT, the abundance file is optional. No abundance file was specified and binning was performed using tetranucleotide frequencies only.

Evaluation of the binning performance

For the datasets of known composition (Shakya2013, Gregor2016, ONT), the sensitivity (recall), precision (positive predictive value), accuracy, and F1 score (harmonic mean of sensitivity and precision) were calculated using the confusionMatrix-function from the caret R-package (26). For each taxon, T, the bin, t, with the majority of the sequences of T is identified. “Unbinned” sequences (either too short or not assigned to a bin; only for MaxBin2 and MetaBAT) were assigned to a special bin, “0”. If the majority of T was assigned to this special bin, i.e., t = “0”, the next best bin, t’, was chosen, t = t’. The definitions of true positive (TP), false positive (FP), false negative (FN), and true negative (TN) are as follows:

- TP – # sequences of T assigned to t
- FP – # sequences in t assigned to taxa other than T
- FN – # sequences of T missing from t
- TN – # sequences being neither TP, FP, nor FN.

Using these definitions, the following performance measures were computed:

- Sensitivity = TP/(TP + FN)
- Precision = TP/(TP + FP)
- Accuracy = (TP + TN)/(TP + FP + FN + TN)
- F1 score = 2*TP/(2*TP + FP + FN)

Supplementary Notes - Evaluation on assembled ground truth data

Shakya2013

A total of 30,122 contigs with a minimal length of 500 bp and a total length of 194,366,918 bp were assembled. After taxonomic annotation and filtering (Supplementary Materials), 24,974 contigs with a total length of 179,063,212 bp remained for binning. 45 bins were identified by BusyBee Web while 58 species were included in the dataset. The bins were 65.79%/84.68% (mean/median) complete, had 30.47%/2.70% contamination, and 15.23%/0.00% strain heterogeneity (Supplementary Table 2). The low average completeness values are likely due to the archaeal populations in this dataset; the set of single copy marker genes (“essential genes”) used by BusyBee Web was originally constructed such that they hit only one gene in >= 95% of bacterial genomes (27), i.e., they do not represent archaeal or other non-bacterial microbial genomes. While non-bacterial microbial organisms are clearly relevant in many microbial communities, using a custom set in CheckM skips the genome tree placement step and considerably reduces memory requirements. As these requirements are crucial aspects in the design of a webserver with multiple concurrent users, we used this custom, albeit restricted set, thereby circumventing the prohibitive memory requirement of CheckM. We used the same custom set to compute the degrees of completeness, contamination and strain heterogeneity

for MaxBin2 and MetaBAT (Supplementary Table 2). MaxBin identified 58 bins and had a higher completeness degree (66.48%/85.59%) as well as lower contamination degree (7.83%/2.25%) than BusyBee Web. MetaBAT identified 63 bins, which were overall less complete (56.07%/71.17%) compared to those returned by BusyBee Web and MaxBin2. The contamination degree of 9.01%/0.90% was lower than that of BusyBee Web but higher than the contamination degree of MaxBin2.

Comparing the binning results to the ground truth information, i.e., each sequence is a priori assigned to a distinct microbial species, sensitivity values of BusyBee Web were 80.73%/85.84% (Supplementary Table 3). The mean precision was relatively low (57.72%), while the median value was 71.99%. The F1 score was 59.30%/70.02%. The low precision could be explained by some bins containing sequences from distinct organisms. Inspection of the 2D embedding revealed that some bins represent 2 – 3 organisms because the automated clustering considered them as one population (Supplementary Figure 2). However, a user-driven inspection would likely recognize several of the contaminated bins, even in the absence of annotation information (bin quality or taxonomy). These bins could then be further refined. Moreover, sequence chunks can be used instead of the full-length sequences, thereby improving the binning performance (see below). BusyBee Web was more sensitive than MaxBin2 and MetaBAT (80.73%/85.84% vs. 70.35%/71.58% vs. 61.06%/60.00%) which is in line with the CheckM-based results. While the median precision of BusyBee Web was higher than that of MaxBin2 (71.99% vs. 57.09%), the median precision of MetaBAT was the highest of the tested tools (99.10%). In terms of F1 scores the mean of MetaBAT was higher than for the other two tools, yet the median was comparable to that of BusyBee Web (70.93% vs. 70.02%; Supplementary Table 3).

Executing BusyBee Web on sequence chunks with a chunk length of 3 kbp, i.e., non-overlapping, consecutive subsequences of a maximum length of 5,999 bp, and a compression of 1, resulted in the recovery of 60 bins (Figure 2 A) with 55.03%/72.52% (mean/median) completeness, 17.45%/3.60% contamination, and 9.27%/0% strain heterogeneity (Supplementary Table 2). The reduction in mean/median completeness is likely an artefact of the chunk creation process, i.e., single copy marker genes are split and thus cannot be reliably identified by CheckM. MaxBin2 failed to bin the sequence chunks as it was unable to identify seed contigs which is in line with the observed reduction in mean/median completeness. MetaBAT identified 91 bins for the chunked data and had completeness and contamination degrees of 37.92%/21.62% and 7.75%/0.90% respectively. However, substantially improved performance values were observed, in particular for BusyBee Web, when considering the ground truth information rather than the presence of single copy marker genes (Supplementary Table 3). The sensitivity values increased to 91.00%/93.47% and the precision increased to 77.26%/91.49%. While the mean/median F1 score for MetaBAT was higher than when applied to the full-length contigs (66.89%/70.93% vs. 70.73%/80.87%), the use of sequence chunks yielded even higher F1 scores for BusyBee Web (80.19%/90.09%).

Gregor2016

This dataset consisted of 14,393 contigs with a length >= 1 kbp and a total length of 142,556,476 bp. A total of 38 bins was identified by BusyBee Web (Supplementary Figure 4) while 45 species were included in the dataset. The bins were 72.71%/92.79% (mean/median) complete, had 25.53%/2.25% contamination, and 28.36%/4.6% strain heterogeneity (Supplementary Table 4). MetaBAT identified 41 bins with 65.28%/83.78% completeness, 22.65%/0.90% contamination, and 28.23%/0.00% strain heterogeneity. Sensitivity values were 88.17%/91.26% and 71.29%/72.9% at the species level for BusyBee Web and MetaBAT, respectively (Supplementary Table 5). The respective F1 scores were 75.87%/88.68% and 63.79%/68.71%.
Executing BusyBee Web on the sequence chunks with a chunk length of 3 kbp (46,520 sequences) (Supplementary Figure 5) resulted in 50 bins with 57.93%/82.88% (mean/median) completeness, 16.88%/4.50% contamination, and 15.73%/0.00% strain heterogeneity (Supplementary Table 4). MetaBAT returned 54 bins with 43.99%/34.24% completeness, 22.76%/2.70% contamination, and 12.94%/0.00% strain heterogeneity. The reduced completeness degrees compared to the full-length sequences are likely artefacts of the chunk creation process, as discussed for the Shakya2013 dataset. Comparing the results against the ground truth, BusyBee Web's sensitivity increased to 94.31%/96.06% and the precision to 81.38%/96.35% when using sequence chunks (Supplementary Table 5). The sensitivity and precision of MetaBAT were found to be 77.19%/77.49% and 64.39%/82.65%, respectively. The F1 score of BusyBee Web was 84.77%/95.21% compared to 63.02%/71.94% for MetaBAT.

Supplementary Figures

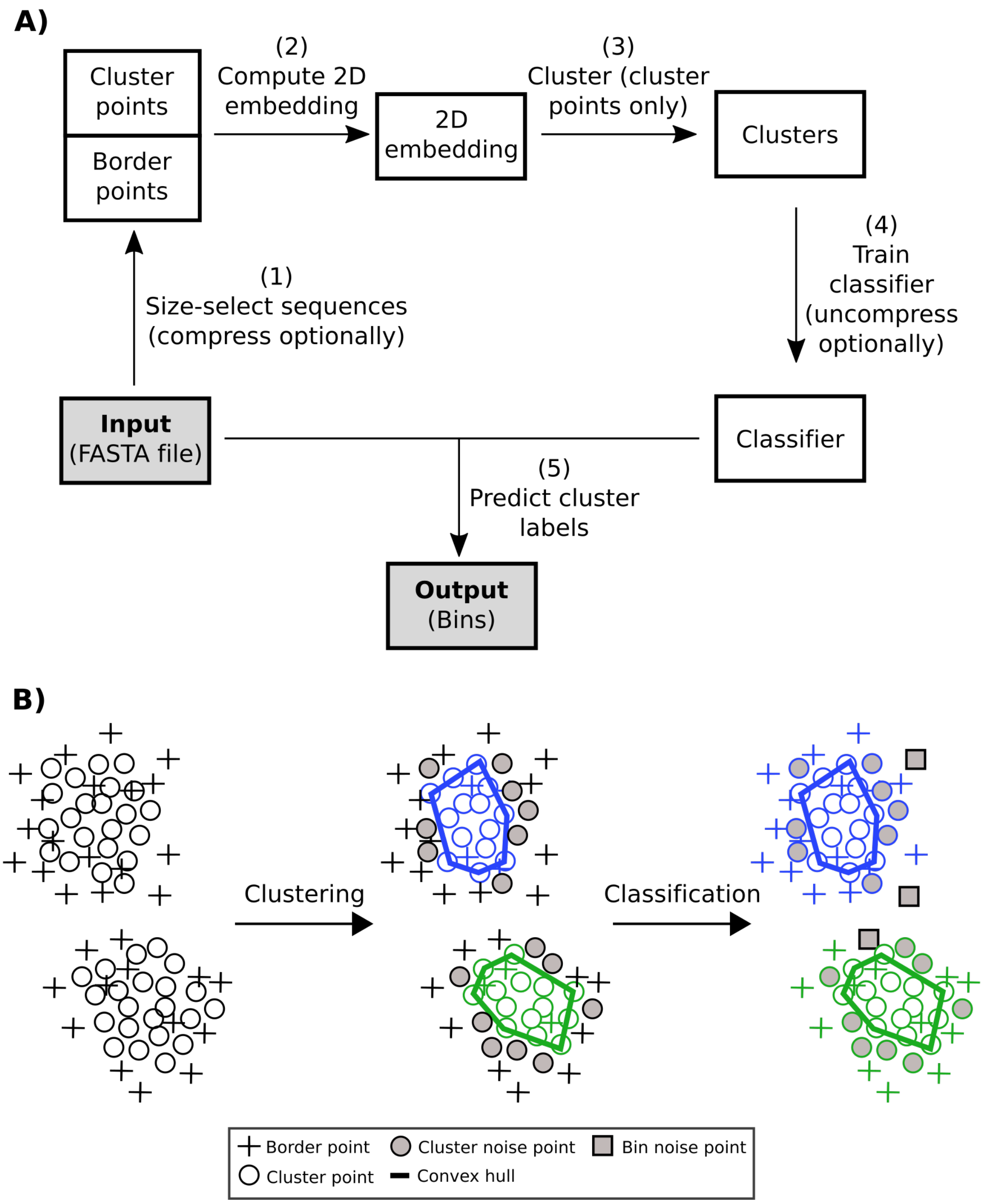

Supplementary Figure 1. Graphical explanation of bootstrapped supervised binning (BSB). A) Workflow of BSB. Population-level genomic bins are defined using a combination of unsupervised (clustering) and supervised (classification) machine learning approaches. The input sequences are first embedded into a 2D space and then clustered. The resulting cluster labels are used as training labels and the trained classifier assigns each input sequences into a distinct bin. Input and output are highlighted by a grey background. B) Illustration of the concepts of border points, cluster points, cluster noise points, convex hull, and bin noise points. Border points and cluster points are defined according to their respective sequence length thresholds (t_b, t_c). The automated clustering, which is applied only to the cluster points only, results in assigned cluster points (cluster points encircled by the convex hull and colored according to their respective cluster, here, blue or green) and cluster noise points (cluster points outside of the convex hull, grey-fill). The border points help pushing clusters further apart from neighboring clusters during the 2D embedding step and are ignored during the clustering process. The classification step assigns labels to all of the points, i.e., regardless whether they are border points, cluster points etc. The resulting point groups (representing population-level sequences groups) are called bins. Depending on the specified probability threshold, bin noise points might be created, i.e., points for which the maximum class-probability was below the specified threshold.

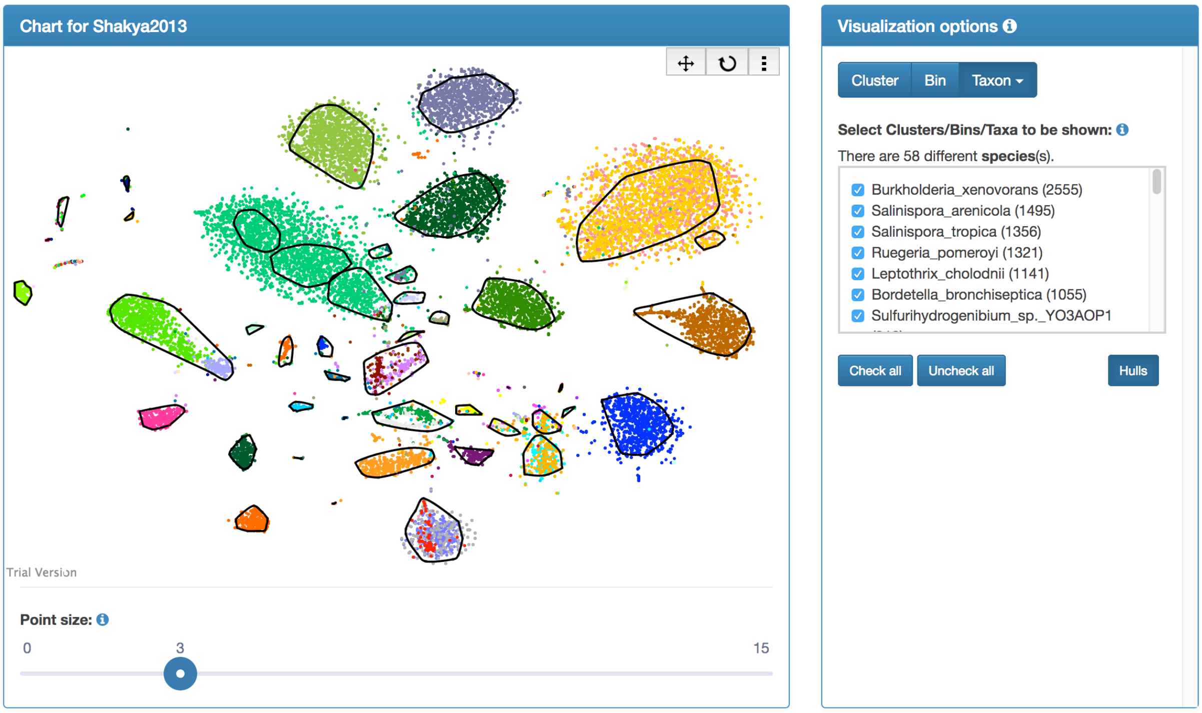

Supplementary Figure 2. BusyBee Web screenshot of the 2D embedding for the Shakya2013 metagenomic dataset. Point colors are according to the taxonomic assignment at the species level.

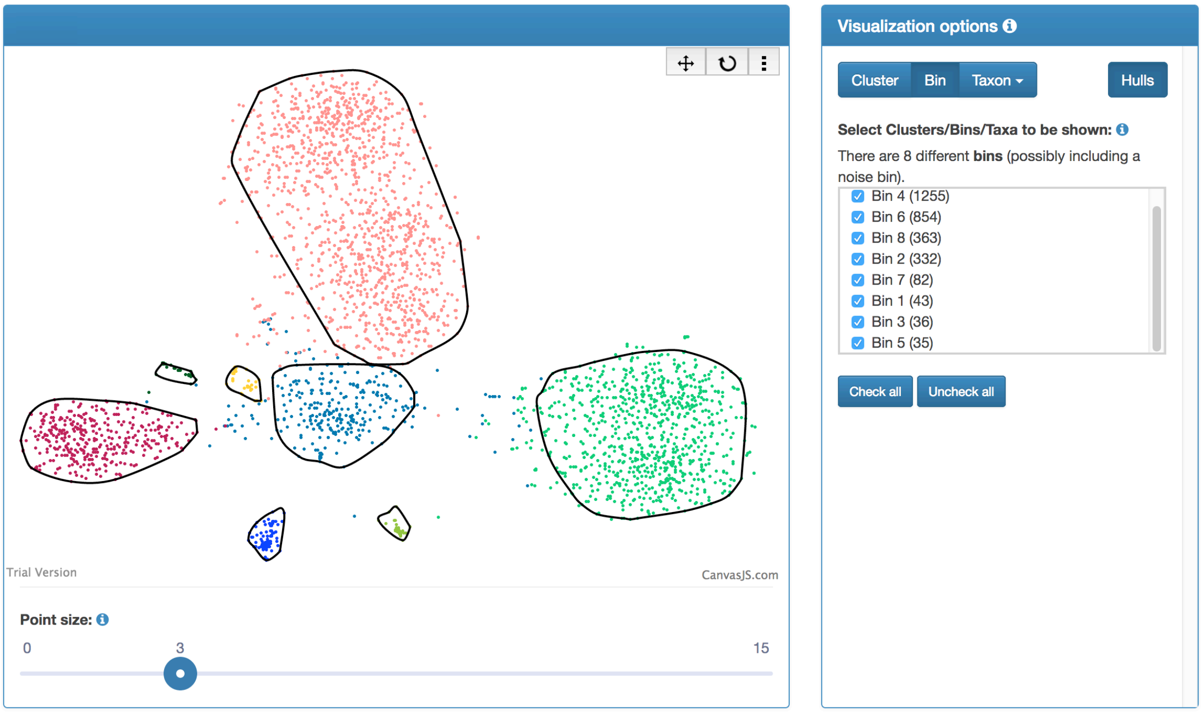

Supplementary Figure 3. Influenza A virus ONT-based subsampled reads. Points are colored according to their assigned bin.

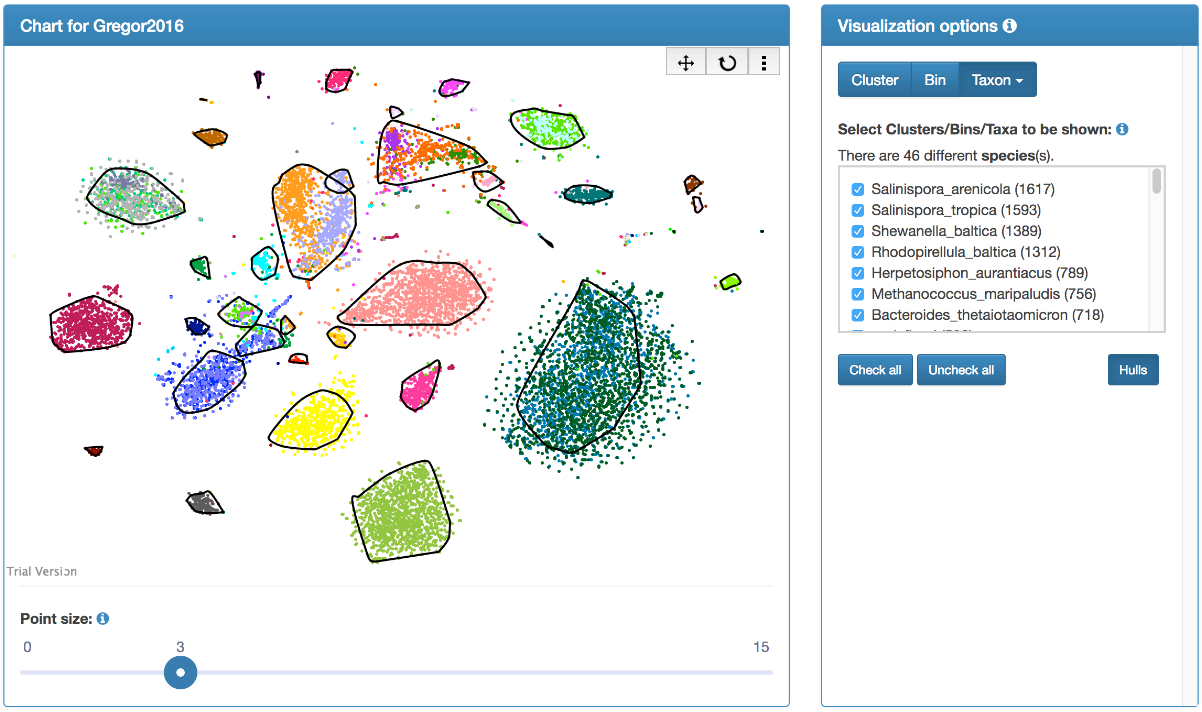

Supplementary Figure 4. BusyBee Web screenshot of the 2D embedding for the Gregor2016 metagenomic dataset. Point colors are according to the taxonomic assignment at the species level.

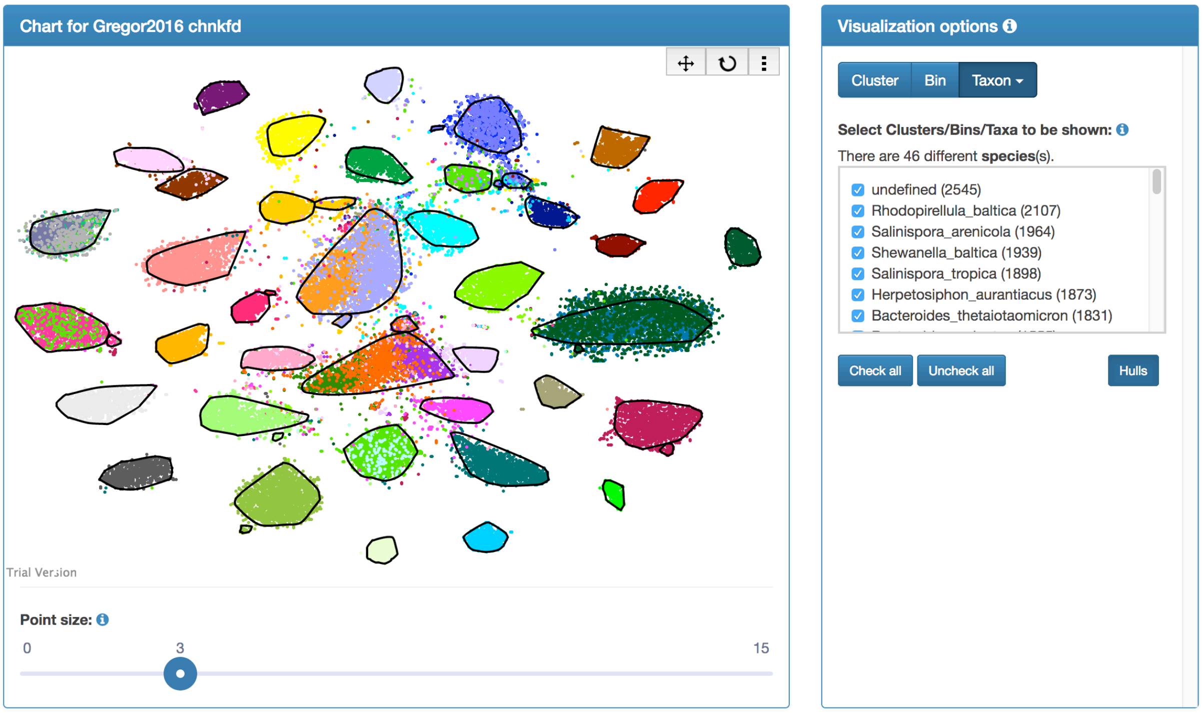

Supplementary Figure 5. BusyBee Web screenshot of the 2D embedding for the Gregor2016 metagenomic dataset and a chunk-length of 3 kbp. Point colors are according to the taxonomic assignment at the species level.

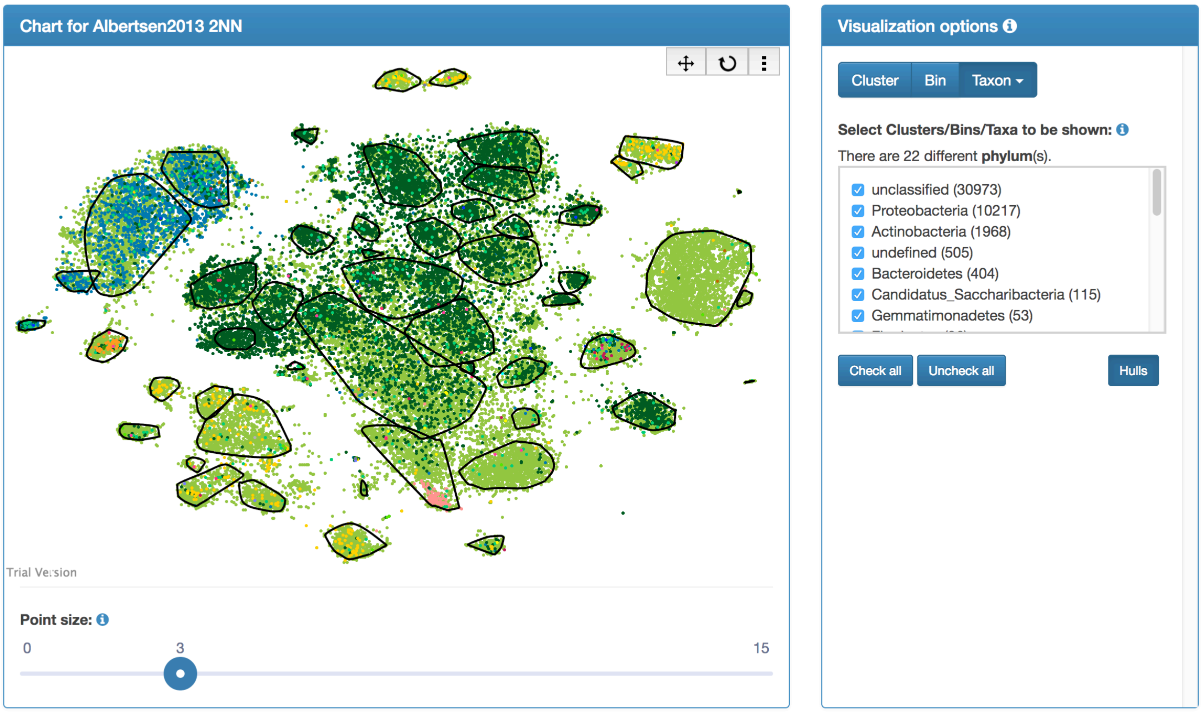

Supplementary Figure 6. BusyBee Web screenshot of the 2D embedding for the large-scale Illumina dataset. A minimum sequence length of 1 kbp and a compression of 2 (“2NN”) were used. Point colors are according to the taxonomic assignment at the phylum level.

Supplementary Tables

Supplementary Table 1. Illumina-based, Pacific Biosciences (PacBio)-based, and ONT-based datasets.

**Dataset**	**Sample**	**Source**
**Small-scale Illumina**	Female infant fecal	http://ggkbase.berkeley.edu/NICU-Micro/download?type=contigs (login required)
**Large-scale Illumina**	Activated sludge bioreactor	ftp://ftp.ncbi.nlm.nih.gov/sra/wgs_aux/AP/MI/APMI01/APMI01.1.fsa_nt.gz
**PacBio**	Biogas reactor microbiome	ftp://ftp.sra.ebi.ac.uk/vol1/fastq/SRR242/006/SRR2420276/SRR2420276_1.fastq.gz
**ONT**	*Agrobacterium tumefaciens*	ftp://ftp.sra.ebi.ac.uk/vol1/fastq/SRR319/005/SRR3191595/SRR3191595.fastq.gz
**ONT**	*Influenza A virus*	ftp://ftp.sra.ebi.ac.uk/vol1/ERA462/ERA462729/oxfordnanopore_native/all_2d_reads.fasta.tar.gz
**ONT**	*Francisella* sp.	ftp://ftp.sra.ebi.ac.uk/vol1/fastq/SRR159/000/SRR1596330/SRR1596330.fastq.gz
**ONT**	*Escherichia coli*	ftp://ftp.sra.ebi.ac.uk/vol1/ERA411/ERA411499/oxfordnanopore_native/flowcell_20_LomanLabz_PC_Ecoli_K12_R7.3.tar
**ONT**	*Enterobacter kobei*	ftp://ftp.sra.ebi.ac.uk/vol1/fastq/ERR134/005/ERR1341575/ERR1341575.fastq.gz
**ONT**	*Salmonella enterica*	ftp://ftp.sra.ebi.ac.uk/vol1/ERA415/ERA415420/oxfordnanopore_native/flowcell6.tgz
**ONT**	*Staphylococcus aureus*	ftp://ftp.ncbi.nlm.nih.gov/sra/Submissions/SRA276/SRA276633/provisional/SRZ101435/20150409_MN02190_FAA36135_BOWDEN04_MdC_Brut_v5.tar.gz

Supplementary Table 2. Shakya2013 completeness, contamination, and strain heterogeneity results based on CheckM. Mean and median percent values are shown for the tested tools and full-length sequences or sequence chunks (chunk length of 3 kbp). A compression of 1 was used for BusyBee Web and the sequence chunks. MaxBin2 returned no bins for the chunked sequences, indicated by "N/A".

		BusyBee Web		MetaBAT		MaxBin2	
		**Mean**	**Median**	**Mean**	**Median**	**Mean**	**Median**
**Completeness**	**Full-length**	65.79	84.68	56.07	71.17	66.48	85.59
	**Chunked**	55.03	72.52	37.92	21.62	N/A	N/A
**Contamination**	**Full-length**	30.47	2.70	9.01	0.90	7.83	2.25
	**Chunked**	17.45	3.60	7.75	0.90	N/A	N/A
**Strain heterogeneity**	**Full-length**	15.23	0.00	10.86	0.00	12.27	0.00
	**Chunked**	9.27	0.00	7.24	0.00	N/A	N/A

Supplementary Table 3. Shakya2013 species-level binning performance based on ground truth. Mean and median percent values of sensitivity, precision, accuracy, and F1 score are shown for the tested tools and full-length sequences or sequence chunks (chunk length of 3 kbp). A compression of 1 was used for BusyBee Web and the sequence chunks. MaxBin2 returned no bins for the chunked sequences, indicated by "N/A".

		BusyBee Web		MetaBAT		MaxBin2	
		**Mean**	**Median**	**Mean**	**Median**	**Mean**	**Median**
**Sensitivity**	**Full-length**	80.73	85.84	61.06	60.00	70.35	71.58
	**Chunked**	91.00	93.47	69.79	73.81	N/A	N/A
**Precision**	**Full-length**	57.72	71.99	81.60	99.10	59.76	57.09
	**Chunked**	77.26	91.49	80.69	97.92	N/A	N/A
**Accuracy**	**Full-length**	98.37	99.43	98.63	99.78	98.83	99.56
	**Chunked**	99.00	99.61	98.59	99.50	N/A	N/A
**F1**	**Full-length**	59.30	70.02	66.89	70.93	59.80	60.08
	**Chunked**	80.19	90.09	70.73	80.87	N/A	N/A

Supplementary Table 4. Gregor2016 completeness, contamination, and strain heterogeneity results based on CheckM. Mean and median percent values are shown for the tested tools and full-length sequences or sequence chunks (chunk length of 3 kbp).

		BusyBee Web		MetaBAT	
		**Mean**	**Median**	**Mean**	**Median**
**Completeness**	**Full-length**	72.71	92.79	65.28	83.78
	**Chunked**	57.93	82.88	43.99	34.24
**Contamination**	**Full-length**	25.53	2.25	22.65	0.90
	**Chunked**	16.88	4.50	22.76	2.70
**Strain heterogeneity**	**Full-length**	28.36	4.60	28.23	0.00
	**Chunked**	15.73	0.00	12.94	0.00

Supplementary Table 5. Gregor2016 species-level binning performance based on ground truth. Mean and median percent values of sensitivity, precision, accuracy, and F1 score are shown for the tested tools and full-length sequences or sequence chunks (chunk length of 3 kbp).

		BusyBee Web		MetaBAT	
		**Mean**	**Median**	**Mean**	**Median**
**Sensitivity**	**Full-length**	88.17	91.26	71.29	72.93

	**Chunked**	94.31	96.06	77.19	77.49
**Precision**	**Full-length**	73.61	93.55	68.74	93.51
	**Chunked**	81.38	96.35	64.39	82.65
**Accuracy**	**Full-length**	98.23	99.68	97.08	99.40
	**Chunked**	98.96	99.78	95.82	98.92
**F1**	**Full-length**	75.87	88.68	63.79	68.71
	**Chunked**	84.77	95.21	63.02	71.94

Supplementary Table 6. ONT completeness, contamination, and strain heterogeneity results based on CheckM. Mean and median percent values are shown for the tested tools and full-length sequences.

	**BusyBee Web**		**MetaBAT**		**MaxBin2**	
	**Mean**	**Median**	**Mean**	**Median**	**Mean**	**Median**
**Completeness**	1.88	0.00	1.85	0.00	20.27	20.27
**Contamination**	0.86	0.00	0.65	0.00	8.56	8.56
**Strain heterogeneity**	2.48	0.00	3.28	0.00	28.34	28.34

Supplementary Table 7. ONT species-level binning performance based on ground truth. Mean and median percent values of sensitivity, precision, accuracy, and F1 score are shown for the tested tools and full-length sequences.

	**BusyBee Web**		**MetaBAT**		**MaxBin2**	
	**Mean**	**Median**	**Mean**	**Median**	**Mean**	**Median**
**Sensitivity**	84.65	93.13	64.16	67.57	92.78	96.33
**Precision**	97.14	98.98	70.58	97.25	26.58	20.98
**Accuracy**	97.39	98.03	86.38	86.56	55.82	46.96
**F1**	89.00	92.66	58.35	56.92	40.26	34.56

REFERENCES

1. van der Maaten,L. (2014) Accelerating t-SNE using tree-based algorithms. *J. Mach. Learn. Res.*, **15**, 3221–3245.
2. Qiu,Y. and Mei,J. (2016) RSpectra: Solvers for Large Scale Eigenvalue and SVD Problems.
3. Laczny,C.C., Pinel,N., Vlassis,N. and Wilmes,P. (2014) Alignment-free visualization of metagenomic data by nonlinear dimension reduction. *Sci. Rep.*, **4**, 4516.
4. Laczny,C.C., Sternal,T., Plugaru,V., Gawron,P., Atashpendar,A., Margossian,H.H., Coronado,S., der Maaten,L. van, Vlassis,N. and Wilmes,P. (2015) VizBin - an application for reference-independent visualization and human-augmented binning of metagenomic data. *Microbiome*, **3**, 1.
5. Krijthe,J. (2015) Rtsne: T-Distributed Stochastic Neighbor Embedding using Barnes-Hut Implementation.

6. Ester,M., Kriegel,H.-P., Sander,J. and Xu,X. (1996) A density-based algorithm for discovering clusters in large spatial databases with noise. In *Proceedings of the 2nd International Conference on Knowledge Discovery and Data Mining*. AAAI Press, pp. 226–231.
7. Hahsler,M. (2016) dbscan: Density Based Clustering of Applications with Noise (DBSCAN) and Related Algorithms.
8. Killick,R. and Eckley,I. (2013) changepoint: An R Package for changepoint analysis. *Lancaster Univ.*, **58**, 1–15.
9. Liaw,A. and Wiener,M. (2002) Classification and Regression by randomForest. *R News*, **2**, 18–22.
10. Arya,S., Mount,D., Kemp,S.E. and Jefferis,G. (2015) RANN: Fast Nearest Neighbour Search (Wraps Arya and Mount's ANN Library).
11. Shakya,M., Quince,C., Campbell,J.H., Yang,Z.K., Schadt,C.W. and Podar,M. (2013) Comparative metagenomic and rRNA microbial diversity characterization using archaeal and bacterial synthetic communities. *Environ. Microbiol.*, **15**, 1882–99.
12. Xu,H., Luo,X., Qian,J., Pang,X., Song,J., Qian,G., Chen,J. and Chen,S. (2012) FastUniq: A Fast De Novo Duplicates Removal Tool for Paired Short Reads. *PLoS One*, **7**, 1–6.
13. Bankevich,A., Nurk,S., Antipov,D., Gurevich,A.A., Dvorkin,M., Kulikov,A.S., Lesin,V.M., Nikolenko,S.I., Pham,S., Prjibelski,A.D., *et al.* (2012) SPAdes: A New Genome Assembly Algorithm and Its Applications to Single-Cell Sequencing. *J. Comput. Biol.*, **19**, 455–477.
14. Wood,D.E. and Salzberg,S.L. (2014) Kraken: ultrafast metagenomic sequence classification using exact alignments. *Genome Biol.*, **15**, R46.
15. Gregor,I., Dröge,J., Schirmer,M., Quince,C. and McHardy,A.C. (2016) PhyloPythiaS+: a self-training method for the rapid reconstruction of low-ranking taxonomic bins from metagenomes. *PeerJ*, **4**, e1603.
16. Deschamps,S., Mudge,J., Cameron,C., Ramaraj,T., Anand,A., Fengler,K., Hayes,K., Llaca,V., Jones,T.J. and May,G. (2016) Characterization, correction and de novo assembly of an Oxford Nanopore genomic dataset from Agrobacterium tumefaciens. *Sci. Rep.*, **6**, 28625.
17. Wang,J., Moore,N.E., Deng,Y.M., Eccles,D.A. and Hall,R.J. (2015) MinION nanopore sequencing of an influenza genome. *Front. Microbiol.*, **6**, 1–7.
18. Karlsson,E., Lärkeryd,A., Sjödin,A., Forsman,M. and Stenberg,P. (2015) Scaffolding of a bacterial genome using MinION nanopore sequencing. *Sci. Rep.*, **5**, 11996.
19. Loman,N.J., Quick,J. and Simpson,J.T. (2015) A complete bacterial genome assembled de novo using only nanopore sequencing data. *Nat. Methods*, **12**, 733–5.
20. Quick,J., Ashton,P., Calus,S., Chatt,C., Gossain,S., Hawker,J., Nair,S., Neal,K., Nye,K., Peters,T., *et al.* (2015) Rapid draft sequencing and real-time nanopore sequencing in a hospital outbreak of Salmonella. *Genome Biol.*, **16**, 114.
21. Judge,K., Hunt,M., Reuter,S., Tracey,A., Quail,M.A., Parkhill,J. and Peacock,S.J. (2016) Comparison of bacterial genome assembly software for MinION data and their applicability to medical microbiology. *Microb. Genomics*, **2**.
22. Bradley,P., Gordon,N.C., Walker,T.M., Dunn,L., Heys,S., Huang,B., Earle,S., Pankhurst,L.J., Anson,L., de Cesare,M., *et al.* (2015) Rapid antibiotic-resistance predictions from genome

sequence data for Staphylococcus aureus and Mycobacterium tuberculosis. *Nat. Commun.*, **6**, 10063.

23. Wu,Y.W., Simmons,B.A. and Singer,S.W. (2015) MaxBin 2.0: An automated binning algorithm to recover genomes from multiple metagenomic datasets. *Bioinformatics*, **32**, 605–607.
24. Kang,D.D., Froula,J., Egan,R. and Wang,Z. (2015) MetaBAT, an efficient tool for accurately reconstructing single genomes from complex microbial communities. *PeerJ*, **3**, e1165.
25. Li,H., Handsaker,B., Wysoker,A., Fennell,T., Ruan,J., Homer,N., Marth,G., Abecasis,G., Durbin,R. and 1000 Genome Project Data Processing Subgroup (2009) The Sequence Alignment/Map format and SAMtools. *Bioinformatics*, **25**, 2078–2079.
26. Kuhn,M. (2016) caret: Classification and Regression Training.
27. Dupont,C.L., Rusch,D.B., Yooseph,S., Lombardo,M.-J., Richter,R.A., Valas,R., Novotny,M., Yee-Greenbaum,J., Selengut,J.D., Haft,D.H., *et al.* (2012) Genomic insights to SAR86, an abundant and uncultivated marine bacterial lineage. *ISME J.*, **6**, 1186–1199.
